# Monitoring Molecular Weight Changes during Technical Lignin Depolymerization by Operando Attenuated Total Reflectance Infrared Spectroscopy and Chemometrics

**DOI:** 10.1002/cssc.202101853

**Published:** 2021-11-23

**Authors:** Khaled N. M. Khalili, Peter de Peinder, Jacqueline Donkers, Richard J. A. Gosselink, Pieter C. A. Bruijnincx, Bert M. Weckhuysen

**Affiliations:** ^1^ Inorganic Chemistry and Catalysis Debye Institute for Nanomaterials Scienceyy Utrecht University Universiteitsweg 99 3584 CG Utrecht The Netherlands; ^2^ Organic Chemistry and Catalysis Debye Institute for Nanomaterials Science Utrecht University Universiteitsweg 99 3584 CG Utrecht The Netherlands; ^3^ VibSpec Haaftenlaan 28 4006 XL Tiel The Netherlands; ^4^ Wageningen Food & Biobased Research Bornse Weilanden 9 6708 WG Wageningen The Netherlands

**Keywords:** ATR-IR spectroscopy, chemometrics, lignin valorization, operando spectroscopy, size exclusion chromatography

## Abstract

Technical lignins are increasingly available at industrial scale, offering opportunities for valorization, such as by (partial) depolymerization. Any downstream lignin application requires careful tailoring of structural properties, such as molecular weight or functional group density, properties that are difficult to control or predict given the structure variability and recalcitrance of technical lignins. Online insight into changes in molecular weight (*M*_w_), to gauge the extent of lignin depolymerization and repolymerization, would be highly desired to improve such control, but cannot be readily provided by the standard ex‐situ techniques, such as size exclusion chromatography (SEC). Herein, operando attenuated total reflectance infrared (ATR‐IR) spectroscopy combined with chemometrics provided temporal changes in *M*_w_ during lignin depolymerization with high resolution. More specifically, ex‐situ SEC‐derived *M*_w_ and polydispersity data of kraft lignin subjected to aqueous phase reforming conditions could be well correlated with ATR‐IR spectra of the reaction mixture as a function of time. The developed method showed excellent regression results and relative error, comparable to the standard SEC method. The method developed has the potential to be translated to other lignin depolymerization processes.

## Graphical abstract

**Weight watching**: An operando attenuated total reflectance infrared (ATR‐IR) spectroscopy‐chemometrics method that correlates size exclusion chromatography‐derived molecular weight (*M*_w_) and polydispersity (PD) measurements of a kraft lignin depolymerization process with the corresponding operando ATR‐IR spectra is developed. The approach is demonstrated here for aqueous phase reforming of kraft lignin but is anticipated to be applicable more generally to various lignin depolymerization processes in biorefinery to provide information of *M*_w_ of lignin in real time.

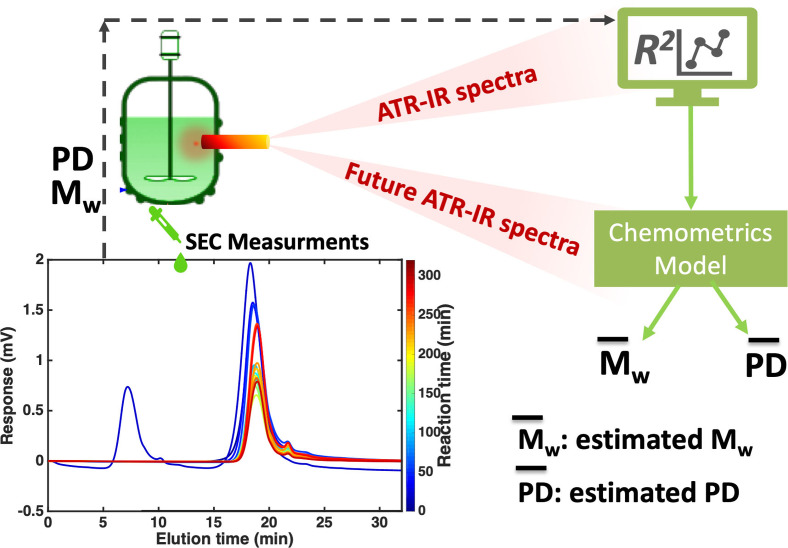

## Introduction

Lignin, an aromatic biopolymer that gives plants their structural integrity, holds considerable potential for the production of renewable chemicals and materials.[^1^, ^2^, ^3^, ^4^, ^5^, ^6^, ^7^, ^8^, ^9^, ^10^] Several biorefining operations, including the pulp and paper industry and 2nd‐generation bio‐ethanol plants generate streams of so‐called technical lignins. Some of these technical lignins are available at scale in isolated form. As the supply of these is anticipated to increase, economic valorization of such streams beyond energy recovery is necessary, for example, to renewables‐based materials or biobased chemical building blocks. The latter strategy would require (partial) lignin depolymerization as a first step.^[11]^

Lignin depolymerization specifically aims to reduce the feed's molecular weight (*M*_w_) by breaking the inter‐unit linkages to ultimately produce low‐*M*_w_ aromatics, such as monomers that can serve as pure platform molecules for further upgrading[^1^, ^2^, ^7^] or lower‐ to intermediate‐*M*_w_ oligomers that can be used as mixture in various applications.^[12]^ While native lignins are rich in relatively easily cleavable aryl−alkyl ether β‐O‐4 bonds, technical lignins are typically characterized by highly condensed and highly variable structures that depend not only on botanical origin but also strongly on the chemical isolation method, with kraft, alkali or organosolv processes being typical examples.[^7^, ^13^, ^14^] Technical lignins have significantly less cleavable ether bonds and contain more carbon–carbon bonded units.^[2]^ The latter are the result of condensation reactions between soluble monomeric and oligomeric compounds originating from bond cleavage in the native lignin;^[15]^ such C−C bond forming processes thus lead to molecular weight increases in the lignin product. Various approaches, including oxidation, hydrodeoxygenation, pyrolysis, hydrolysis, and hydrogenolysis reactions, have been explored to (partially) depolymerize technical lignins.[^16^, ^17^, ^18^, ^19^, ^20^, ^21^, ^22^] Base‐catalyzed depolymerization of lignin using sodium hydroxide proved to be an effective strategy to depolymerize technical lignin,^[23]^ for example. The alkaline conditions ensure good solubilization of the lignin feedstock and generated fragments. Base‐catalyzed depolymerization followed by catalytic hydrodeoxygenation has also been thoroughly investigated to depolymerize lignin into gasoline‐range aromatic fuels.[^24^, ^25^, ^26^] For example, Beckham and co‐workers depolymerized kraft lignin and other technical lignins into low‐molecular‐weight, water‐soluble species with relatively high yields.^[23]^ Aqueous phase reforming (APR) and liquid phase reforming (LPR) of technical lignins can also be performed under alkaline conditions, typically producing moderate to good monomer yields under somewhat milder temperatures and pressures.[^11^, ^22^, ^27^] Under APR and LPR conditions both ether and, to some extent, C−C bond cleavage can occur,^[15]^ both of which can lead to molecular weight reduction.

Control over and thus knowledge of *M*_w_ and polydispersity (PD) are often central to the valorization of technical lignins. *M*_w_ and PD are key parameters for lignin applications in materials synthesis, indicating functionality, homogeneity, and viscosity regimes, as well as valuable indicators of the extent of overall lignin depolymerization when small chemical building blocks are targeted. Most commonly, size exclusion chromatography (SEC) is used as standard technique to determine the molecular weight of lignin. However, *M*_w_ analysis by SEC can be tedious, involving continuous calibration, careful sample preparation, and lengthy data acquisition. Furthermore, SEC only offers ex‐situ analysis, limiting sampling frequency, possibly introducing sampling effects, and temporally separating process and analysis. In‐situ analysis of *M*_w_ and PD changes would of course be highly desirable as this could address the issues listed above and would allow one to deal with lignin‐specific issues of batch‐to‐batch and process‐dependent differences in lignin structure and, hence, reactivity. Therefore, an alternative, simple, non‐destructive, and fast analytical technique that can reliably supply information on *M*_w_ and PD in real time would offer great advantages. Fourier‐transform infrared (FT‐IR) spectroscopy combined with multivariate regression is a powerful approach to provide quantitative structural information and in principle could supply this information since the FT‐IR spectra contain a wealth of chemical information on the lignin sample, including relative abundance of linkages and functional groups and even, somewhat surprisingly, information on molecular weight. Indeed, partial least squares (PLS) regression combined with FT‐IR spectroscopy has already been used for quantification of some structural units and functional groups in lignin,[^28^, ^29^] and the characterization of whole biomass.[^30^, ^31^, ^32^, ^33^, ^34^, ^35^, ^36^] Recently, it was reported that *M*_w_ values and inter‐unit linkage abundances could also be obtained for a wide range of technical lignins directly from their attenuated total reflectance (ATR)‐IR spectra by chemometrics.^[37]^ The FT‐IR spectra were simply acquired from the solid lignin powder under ambient conditions using a standard ATR‐IR accessory. This demonstrates that information on *M*_w_ is in principle tractable from FT‐IR spectra and poses the question if this method for off‐line solid sample analysis could be extended to on‐line monitoring of a lignin depolymerization reaction, for example, as part of a prospective biorefinery operation.

Reliably extracting *M*_w_ information from operando ATR‐IR spectroscopy measurements comes with considerable analytical challenges, given that the depolymerization reactions are often operated at elevated temperature and pressure and are run in IR light‐absorbing solvents, including water. These contributions to the ATR‐IR spectra must be adequately addressed beforehand. Previously, we reported on an analytical protocol to deal with such challenges and to acquire high‐quality ATR‐IR spectra, for example, under APR reaction conditions.^[38]^ Here, we now turn to the challenge of acquiring information on the *M*_w_ from the ATR‐IR spectra by multivariate regression, using an ex‐situ SEC *M*_w_ dataset to calibrate the operando spectroscopic method. Hence, a multivariate regression model was developed with the potential to replace off‐line SEC measurements with online operando spectroscopy measurements to monitor changes in the *M*_w_ value of kraft lignin.

## Results and Discussion

### Solvent‐corrected operando spectra

**Figure 1:**
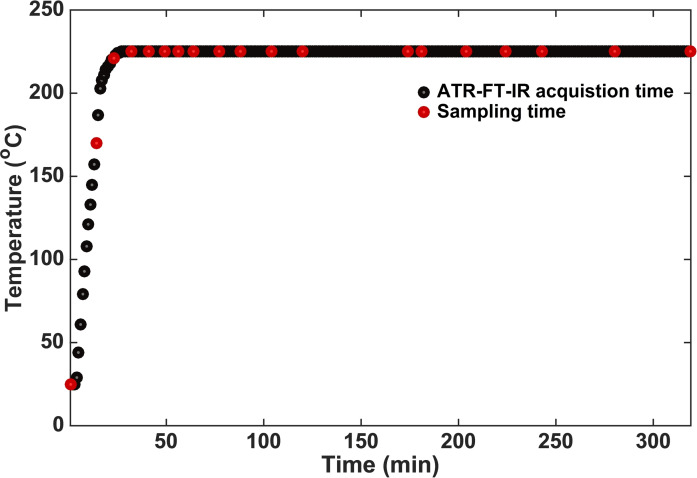
Heating profile vs. operando ATR‐IR spectroscopy acquisition time and sampling time for SEC analysis. The APR reaction of kraft lignin was conducted over a Pt/Al_2_O_3_ catalyst with 3.5 wt% NaOH in a 100 mL Parr autoclave equipped with a bottom‐mounted ATR‐IR accessory.

The depolymerization of kraft lignin was monitored over time under alkaline (3.5 wt% NaOH, pH=13.58) APR conditions (225 °C, 30 bar He) using a commercially available Pt/Al_2_O_3_ catalyst in an autoclave setup equipped with a bottom‐mounted ATR‐IR probe (Figure S1). In total, 19 samples were taken over the course of the reaction for ex‐situ SEC determination of the *M*_w_ and PD values, data needed as input to build the chemometrics model. The first three samples were taken before reaching the set temperature, as depicted in Figure 1.

**Figure 2:**
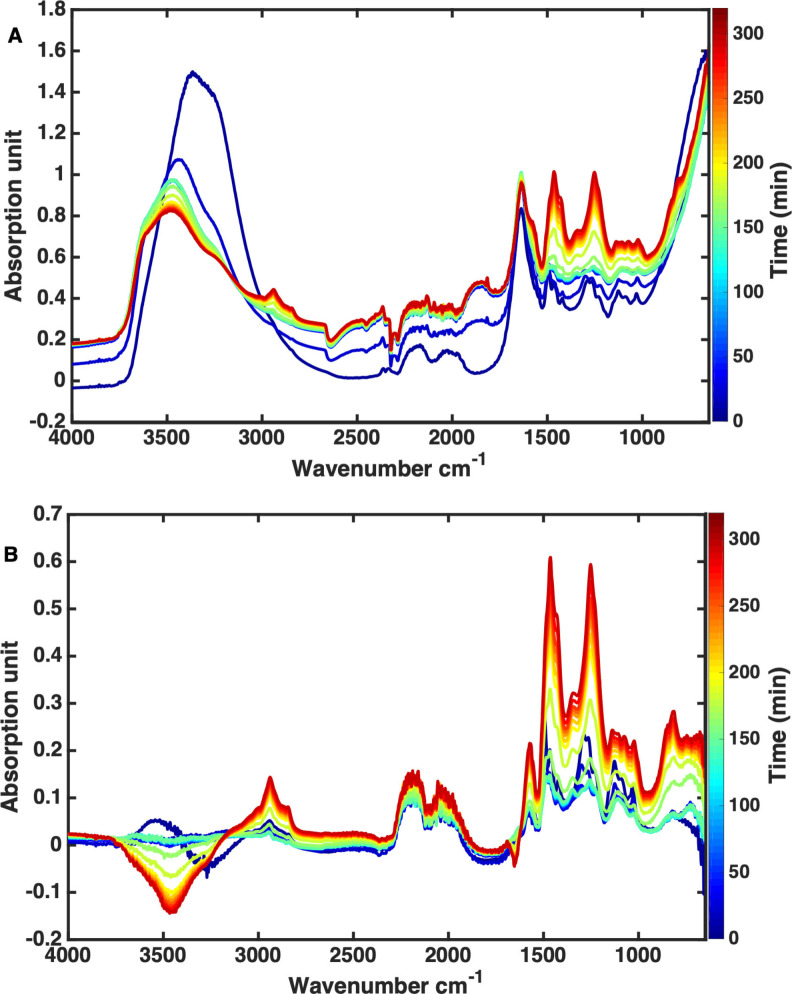
(A) As‐measured operando ATR‐IR spectra, expressed as absorption, for the 19 samples acquired throughout the APR reaction for kraft lignin. (B) Solvent‐corrected operando ATR‐IR spectra where the solvent contribution was removed by the single‐beam method.

The 19 operando ATR‐IR spectra obtained during kraft lignin APR that correspond with the 19 samples drawn from the reaction for SEC analysis are shown in Figure 2A. To prepare the operando ATR‐IR spectra for chemometric analysis, interferences due to solvent and temperature contributions, baseline shift, and light scattering due to the presence of solid particles must be corrected. To do so, the operando ATR‐IR spectra were first corrected for background and temperature‐resolved solvent contributions using the temperature‐dependent single‐beam approach reported previously.^[38]^ To this extent, the ATR‐IR spectra acquired in absorption mode were first converted to the corresponding single‐beam ATR‐IR spectra (Figure S2A), using the background spectrum of the empty ATR‐IR probe‐equipped reaction vessel under ambient conditions. The single‐beam operando ATR‐IR reaction spectra were then ratioed against the corresponding single‐beam spectra of the solvent measured at the identical temperatures. The single‐beam temperature‐resolved operando ATR‐IR spectra of the solvent, at the same pH value as in the reaction run, (2.7 wt% aqueous NaOH, pH=13.58) were obtained in a separate experiment (Figure S2.B). The final, solvent‐corrected operando ATR‐IR spectra are shown in Figure 2B. The noisy spectral regions of 4000–3160 (water OH stretching), 2360–1800 (diamond crystal absorption, CO_2_ antisymmetric vibration), and 940–650 cm^−1^ were removed as they adversely affected the modelling, leaving the information‐rich 3160–2360 and 1800–940 cm^−1^ regions for further analysis.

### Towards a predictive model

**Figure 3:**
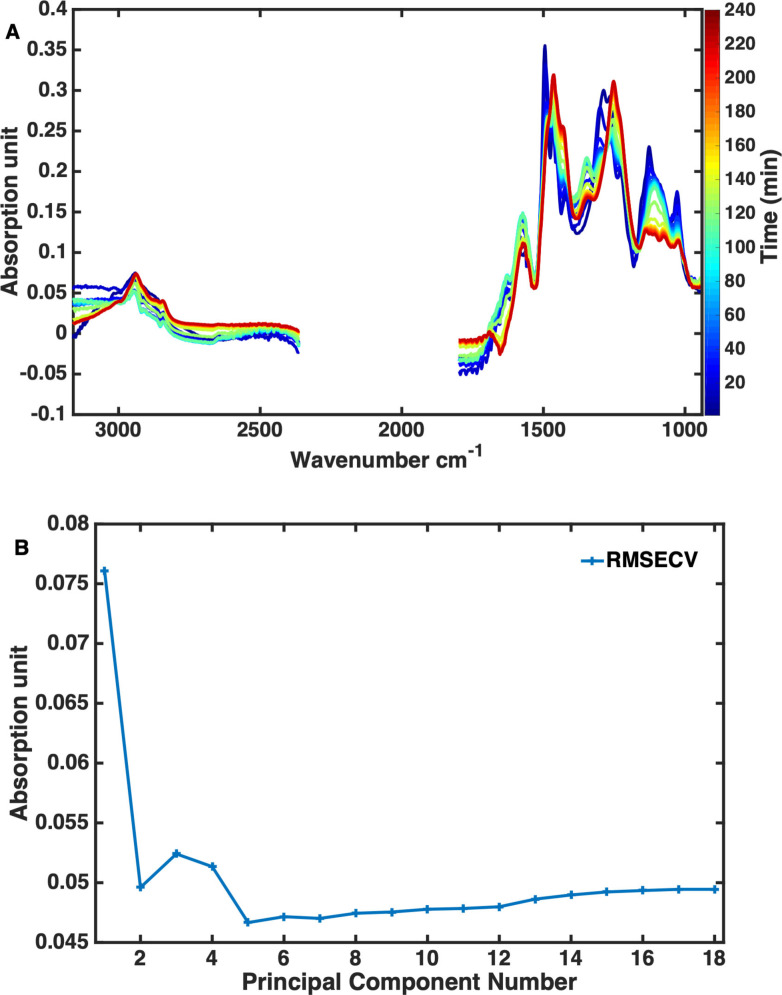
(A) Solvent‐corrected operando ATR‐IR spectra for 19 samples acquired throughout the APR reaction of kraft lignin after MSC pre‐processing on the selected spectral regions. (B) RMSECV of the PCA used to model the operando ATR‐IR spectra of the 19 samples.

Prior to spectral modelling, the operando ATR‐IR spectra were normalized with multiplicative scatter correction (MSC) to adjust for any additive (i. e., baseline, if applicable) and multiplicative scaling effects. Such scaling effects are mainly due to light scattering from solid particles,[^39^, ^40^] that is, either from the solid catalyst or biomass‐derived solids formed during the APR reaction. The selected spectral regions preprocessed with MSC are depicted in Figure 3A. In theory, MSC removes scaling due to interfering systematic effects, such as scattering, while scaling due to properties of interest (such as *M*_w_ variation) is reserved.[^39^, ^40^]

**Figure 4:**
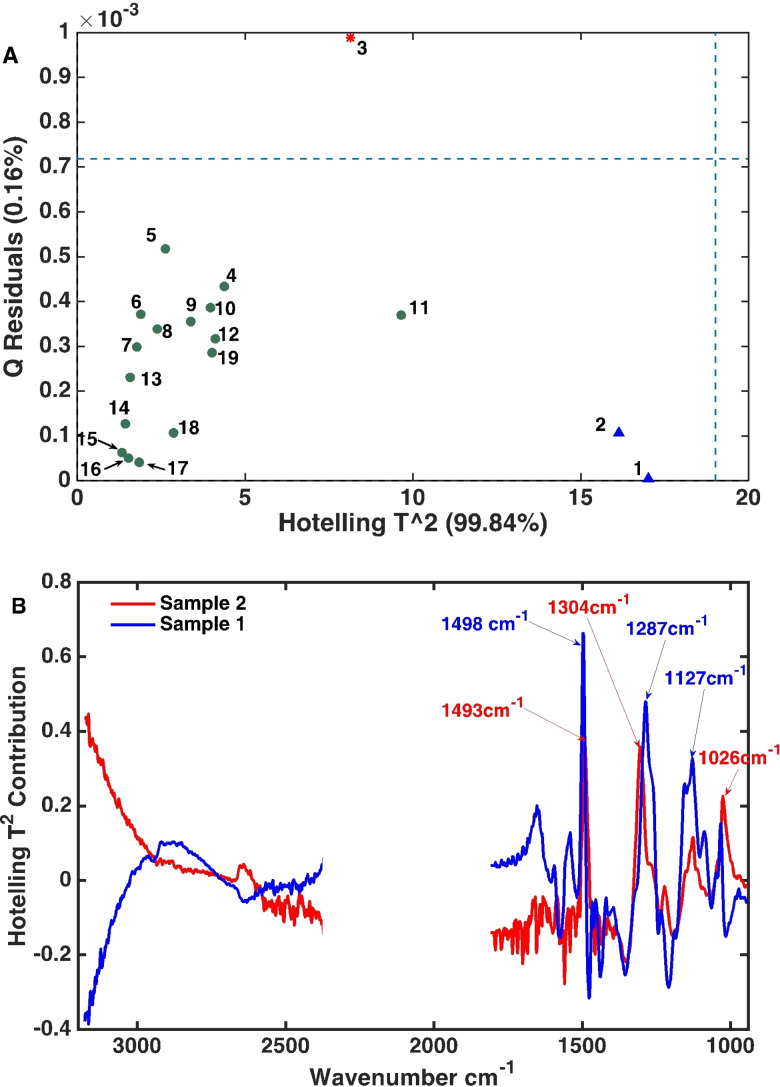
(A) Hotelling's T^2^ vs. the Q residuals (influence plot) of the PCA model built on the MSC pre‐processed ATR‐IR spectra of the APR reaction mixtures. (B) Scores contribution plot for the spectra of samples 1 and 2 reveals the vibrational modes that dominate the operando ATR‐IR spectra.

The normalized ATR‐IR spectra were first explored with a principal component analysis (PCA) model. The model was built with five principal components (PCs) to minimize the root mean square error of cross validation (RMSECV), resulting in an explained variance of 99.84 % (Figure 3B). The influence plot (Figure 4A) shows the spectrum of sample 3 to have more residuals than the other spectra. Notably, when only four PCs were used instead, the spectrum of sample 3 did not produce higher residuals. As the 5th PC explained only 0.19 % of the variance in the data, it would as such not be expected to have significant leverage if kept in the model and was omitted. As evident from the relatively high Hotelling's *T* ^2^ in the influence plot, the ATR‐IR spectra of samples 1 and 2 are the most different. The Hotelling's *T* ^2^ statistic can be viewed as an extension of the *t*‐test^[44]^ and is a common diagnostic tool to detect outliers especially when automation of outlier detection is needed, such as in online process monitoring. Hence, a spectrum that is very different from the bulk of the measured spectra will have extreme scores and thus a high Hotelling's *T* ^2^. In this case, the spectra of samples 1 and 2 should, however, not be considered as outliers as these are acquired at the beginning of reaction and with temperatures very different from the bulk set of spectra. The relatively extreme scores obtained for the spectra of samples 1 and 2 can be understood from looking at the scores contribution plot (Figure 4B).

The two spectra had relatively high contributions from kraft lignin vibrations, that is, aromatic skeletal vibrations (1498 and 1493 cm^−1^), the O−C−O mode in G units (1304 and 1287 cm^−1^)^[41]^ and the C−O−C stretching in the alkyl aryl ether at 1127 cm^−1^.^[42]^ These two spectra are expected to have high contributions from such bonds as they were acquired at early reaction times. The spectra of samples 1–3 were therefore kept in the subsequent regression efforts to check if they have significant leverage on the performance of the constructed model.

**Figure 5:**
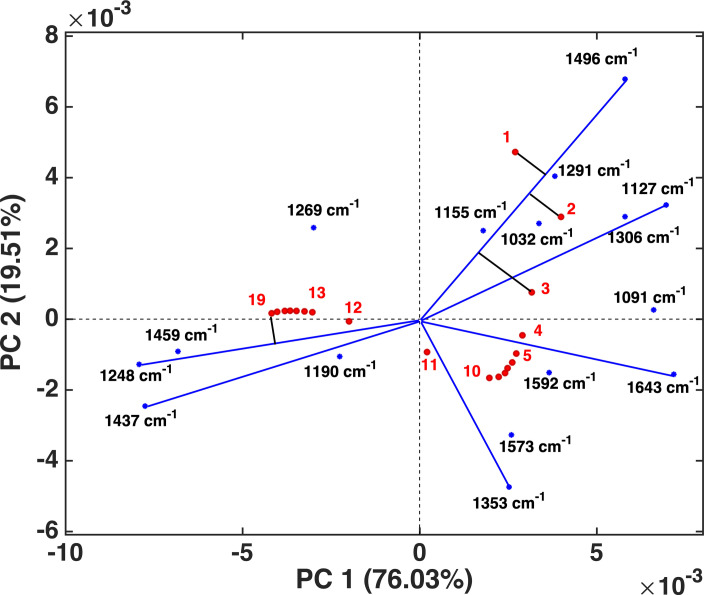
Biplot of PC1 and PC2 showing the projection of samples (red) and the important infrared bands (blue). Lines have been added only for the most important bands.

In general, the MSC‐corrected ATR‐IR spectra (Figure 3A) provided insight into the structural changes that occur during the APR reaction as a function of time. For example, lignin vibrations in the 1150–1000 cm^−1^ region dropped in intensity during the APR reaction, while the band at 1265 shifted to 1250 cm^−1^ and the one at 1287 to 1304 cm^−1^ before being diminished. These spectral changes are also evident from the loadings of the PCA model (see Figure S3). The loadings of the first (PC1) and second (PC2) principal components together explained most (i. e., 95.54 %) of the spectral variation taking place during the reaction and thus provide a compact overview to track these changes. Further insight can be gained from plotting loadings and scores simultaneously in a so‐called biplot (Figure 5), a powerful PCA tool that allows observed variations in the scores to be connected with the source of spectral variation.^[43]^ The lengths of the lines in the biplot reflects the variance of the respective variable.^[44]^

The kraft lignin vibrations at 1032, 1127, 1155, 1291, 1306, and 1496 cm^−1^ were positively correlated (Figure S3). Looking at the projection of a sample onto a variable line, samples 1–3 have the longest cut points and hence the highest value for that variable^[44]^ for all lignin‐related bands; this is shown in Figure 5 for the 1496 cm^−1^ vibration. This indicates that these samples are relatively richer in the IR bands assigned to kraft lignin, as expected. Upon approaching the end of the APR reaction (i. e., samples 12–19), the samples became richer in the bands at 1459, 1248, and 1437 cm^−1^. For example, the cut points to the 1248 cm^−1^ band show that sample 19 has the strongest contribution from this vibration. This band can be assigned to the aryl−O−C stretching vibration in guaiacol, suggesting that the later spectra are more enriched in low‐*M*_w_ reaction products. These observations agree with ex‐situ gas chromatography (GC) and GC‐mass spectrometry (GC‐MS) analysis (Figure S4), which shows that guaiacol is the major product of the APR reaction resulting from a softwood lignin rich in guaiacyl aromatic units. However, as the monomer yield is limited (4.8 % based on lignin intake) and as the vibrational bands in the fingerprint region contain complex contributions from various vibrational modes,^[41]^ one should be cautious in attributing this trend in the biplot to the formation of guaiacol alone.

**Figure 6:**
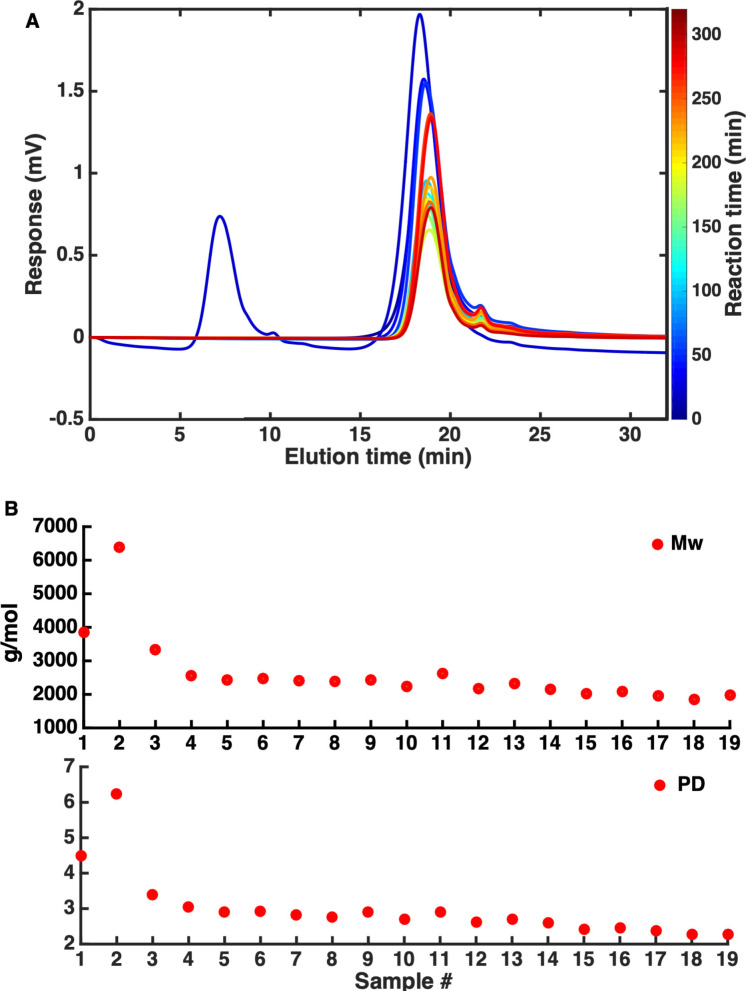
(A) Overlay of the SEC traces of the 19 samples acquired during the APR reaction of kraft lignin. (B) M_w_ and PD values, as determined by SEC, of the 19 samples acquired during the APR reaction.

The results of the ex‐situ SEC measurements are plotted in Figure 6. The SEC samples showed a general shift to lower *M*_w_ as the APR reaction of kraft lignin proceeded. Sample 2, however, showed an anomalous profile, with an additional peak at shorter retention time. The *M*_w_ values extracted from the SEC traces gradually decreased (Figure 6B), eventually plateauing at around 2000 g mol^−1^; the 2nd sample instead showed an increased *M*_w_. The origin of the additional high‐*M*_w_ contribution to sample 2 and its bimodal appearance is unclear and likely an experimental (sampling or contamination) error. As this provided an illustrative real‐world example of how online monitoring models can encounter and should deal with unexpected contributions, this sample was kept in the set, and the effect of this anomaly on the modeling efforts is detailed below.

**Figure 7:**
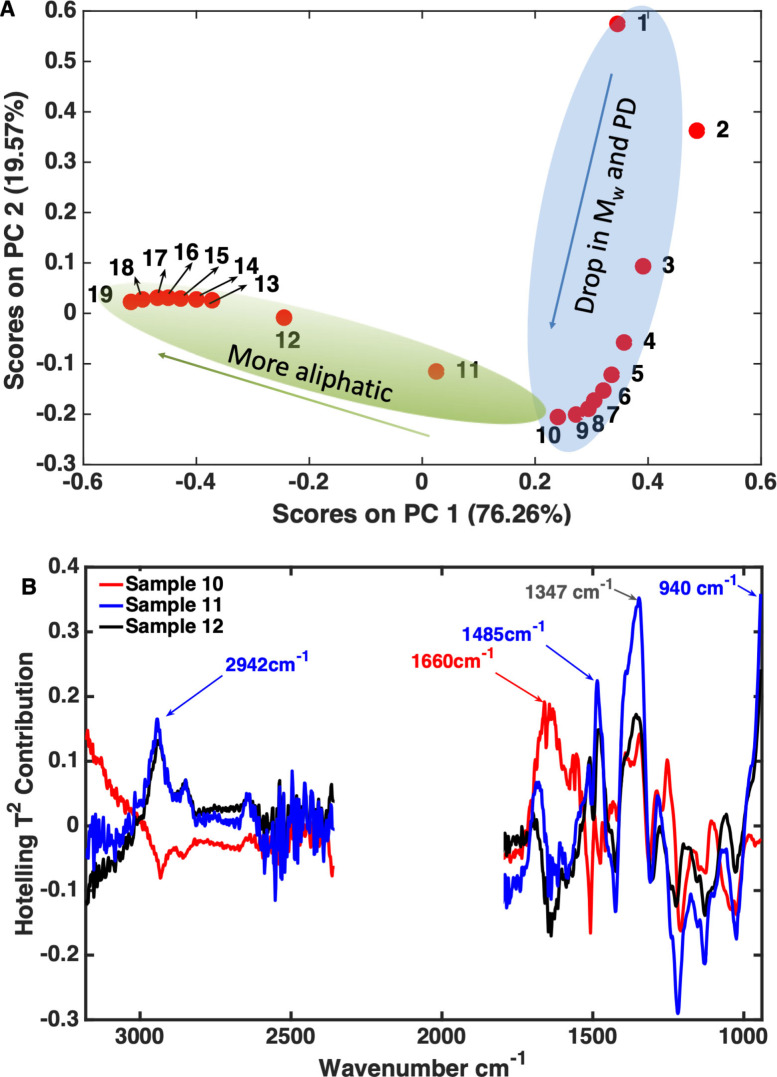
(A) The score plot of PC2 vs. PC1 of the operando ATR‐IR spectra corresponding to the 19 samples acquired during the APR reaction. Arrows show the direction of the trends in the data. The score plot explains 95.82 % of the variance in the spectra. (B) Scores contribution plot of samples 10–12.

An interesting trend in the scores plot, shown in Figure 7A and marked with the blue ellipse, showed that the drop in the scores on PC1 and PC2 was associated with a concomitant decrease in *M*_w_ and PD for samples 1–10, after which the *M*_w_ levels out. Another interesting trend, highlighted in green, shows that the model also picks up the formation of more saturated structures, once the *M*_w_ and PD changes have leveled out, as can be seen going from sample 10 to sample 12 and further. Indeed, for samples 12 and higher the values for *M*_w_ and PD only minimally decreased. Inspection of the scores contribution plots of samples 10–12 (Figure 7B) substantiates this: samples 11 and 12 have stronger contributions of the C−H stretching modes in CH_3_ and CH_2_ (≈2942 cm^−1^), the CH_2_ scissor mode (1472 cm^−1^), and the aromatic skeletal and C−H out of plane (*trans*) bending modes (1485 and 940 cm^−1^, respectively). The latter might be not reliably included, however, being located at the edge of the spectra. Sample 10 showed a large contribution of the 1660 cm^−1^ vibrational mode, which is typical for C=C in alkenes.^[42]^ These olefins in kraft lignin can be found in the cinnamyl alcohol, enol ether, and (β‐1‐ and β‐5‐derived) stilbene structures.^[45]^ Such structures can be hydrogenated under the applied APR conditions. The strong contribution at 1347 cm^−1^ is assigned to an O−H in‐plane bending mode.^[42]^ This closer inspection also led us to remove the 2600–2360 cm^−1^ region in the subsequent regression as it only added noise (see Figures S3 and 7B) and better regression could be obtained by doing so. Finally, that sample 2 is positioned outside the blue ellipse is in line with its *M*_w_ and the PD being higher than those of sample 1.

### Partial least square regression

Next, we built a quantitative model to predict the molecular weight values from the collected operando ATR‐IR spectra by multivariate regression of a training set of the ATR‐IR spectra and by applying a calibration set of SEC *M*_w_ values. To this extent, the obtained data were split into a training set (i. e., two thirds of the samples) and a validation set (i. e., the other third). The validation samples were distributed evenly to cover the whole range of variation. Partial least square (PLS) regression was used for regression. Prior to PLS regression, the ATR‐IR spectra were normalized with MSC. Three PLS regression models were evaluated (further denoted as **A**–**C**), and the best prediction was obtained when non‐logarithmic values were used for *M*_w_ and PD and sample 2 was classified as outlier and excluded (**B**). The results for the PLS regression models are summarized in Table 1.

**Table 1:** Results of PLS regression between average‐weight M_w_ and PD, as determined by SEC of the reaction samples and their corresponding operando ATR‐IR spectra.

Entry	Range		Size^[a]^	LV^[b]^	RMSECV^[c]^		RE^[d]^ [%]		RMSEP^[e]^		Prediction bias		*R* ^2^ (pred.)^[f]^	
	*M*_w_ [g mol^−1^]	PD		*M*_w_ [g mol^−1^]	PD	*M*_w_ [g mol^−1^]	PD	*M*_w_ [g mol^−1^]	PD	*M*_w_ [g mol^−1^]	PD	*M*_w_ [g mol^−1^]	PD	*M*_w_ [g mol^−1^]	PD
A	6380	6.23	19	4	5	391	0.21	4.70	3.21	563	0.62	−121	−0.16	0.81	0.62
B	3851	4.48	18	4	5	391	0.21	7.79	4.69	77	0.09	7	−0.02	0.97	0.97
C^[g]^	3851	4.48	18	3	4	412	0.33	8.65	5.80	161	0.10	−30	−0.02	0.97	0.90

[a] Number of samples included in modeling. [b] LV=latent variable. [c] CV=cross‐validation=leave out one. [d] RE=relative error=RMSECV/range. [e] RMSEP=root mean squared error of prediction. [f] *R* ^2^ (pred.)=coefficient of determination for prediction. [g] Logarithmic values of *M*_w_ and PD were used in this model.

**Figure 8:**
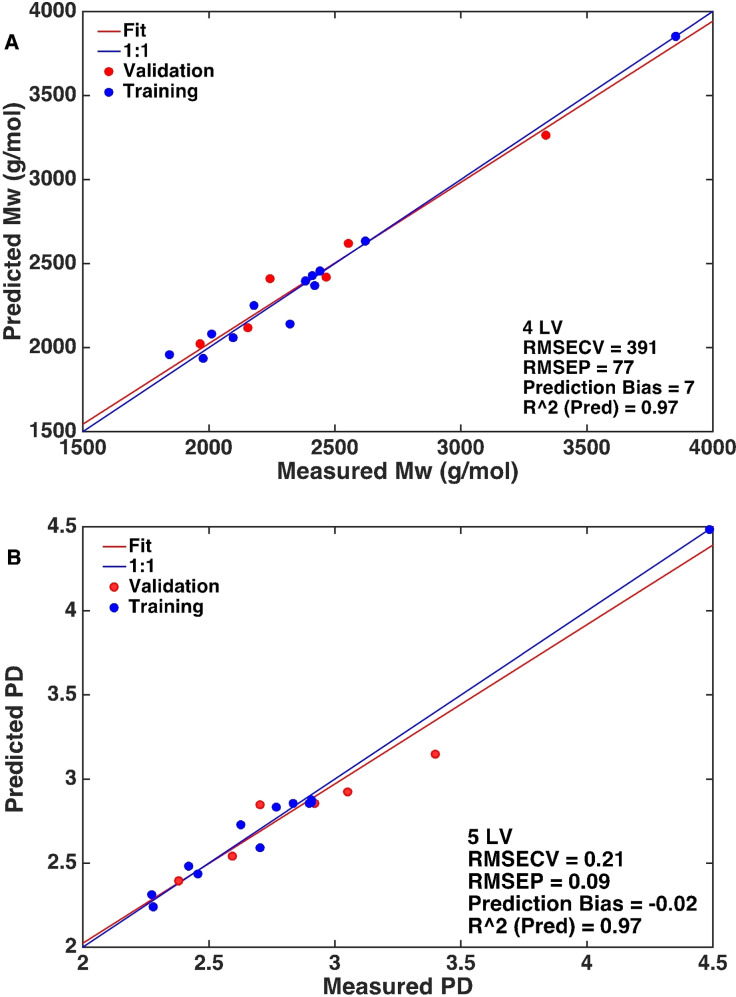
(A) Performance of PLS Model **B** upon prediction of M_w_ along with important statistics. (B) Performance of PLS Model **B** upon prediction of PD along with important statistics.

In PLS Model **A**, the outlier sample 2 was placed in the validation set to check how the model would deal with it. A RMSECV of 391 g mol^−1^ and 0.21 for *M*_w_ and PD, respectively, were obtained in this model. Figure S5A,B shows the performance of the model together with the important statistics. The relatively high RMSECV value for the *M*_w_ was mainly due to sample 11, which was part of the training set. Sample 11 exhibited a relatively high *M*_w_ of 2619 g mol^−1^ compared to sample 10 that had a *M*_w_ of 2242 g mol^−1^. When sample 11 was removed from the training set, the RMSECV value dropped to 240 g mol^−1^; there was, however, no other clear argument to exclude this sample, so it was left in. As inclusion of sample 2 did not lead to a satisfactory model, PLS Model **A** was discarded and PLS Model **B** was established by excluding sample 2. This significantly improved the prediction and produced a regression model with an excellent coefficient of determination *R* ^2^(pred.) of 0.97, small prediction error of 77 g mol^−1^ and 0.09, and small prediction bias of 7 g mol^−1^ and −0.02 for *M*_w_ and PD, respectively. This PLS model showed improved performance in prediction compared to its performance in cross validation. This indicates that this PLS model is better in predicting this particular dataset, but not necessarily better for a future dataset. Figure 8A,B depicts the performance of PLS Model **B** to predict the *M*_w_ and PD values, respectively. PLS Model **C** made use of log_10_(*M*_w_, PD) instead of the non‐logarithmic values, as shown in Figure S6 and Table 1. This model gave slightly less precise cross‐validation results for both *M*_w_ and PD and also proved to be slightly less precise than PLS Model **B** when comparing the root mean squared error of prediction (RMSEP) and prediction bias. This was counter to expectation, as better predictions were expected given that non‐linearity is expected in the SEC molecular weight data used to determine *M*_w_ and *M*_n_ values of the calibration set.^[37]^ For example, standard detection methods (e. g., refractive index) in combination with sulfonated polystyrene standards have shown substantial (non‐linear) errors in the molecular weight results.^[37]^ Apparently this non‐linearity is not sufficiently pronounced in this particular data. Indeed, given its lowest RMSECV and bias, Model **B** (non‐logarithmic values) is the optimum model for future prediction as well as for this dataset. Importantly, this shows, for the first time, that the operando ATR‐IR spectra can be used to obtain information about molecular weights of lignin if the spectra are of sufficient quality. The regression vectors of Model **B** (Figure S7) show which wavenumbers have the greatest influence in predicting the *M*_w_ and the PD; these wavenumbers can be used in further work to optimize the models. Such optimization may require testing different portions of the spectra (variable selection), testing different preprocessing options (1st, 2nd derivative with different smoothing options), and testing different normalization options (MSC, SNV, etc.). Thus, the number of possible models to be tested will rapidly increase. To deal with the hundreds of models to be tested, future studies should focus on automation, for example, through the model optimizer tool available in the PLS_Toolbox.

## Conclusions

Molecular weight (*M*_w_) values of kraft lignin derivatives can be obtained directly from operando attenuated total reflectance infrared (ATR‐IR) spectra recorded in lignin–water mixtures under typical aqueous phase reforming (APR) reaction conditions (i. e., at elevated temperatures and pressures). This is possible provided that a suitable calibration set is available, for example, obtained ex‐situ by size exclusion chromatography (SEC) measurements. A chemometric model, based on partial least squares (PLS) regression, was developed that could correlate and predict time‐dependent *M*_w_ and polydispersity (PD) values with the corresponding operando ATR‐IR spectra of the reaction mixture. Effective correction for the solvent and temperature contributions to the ATR‐IR spectra was key to have operando spectroscopic data of sufficient quality to serve as good predictor variables. Furthermore, principal component analysis (PCA) proved highly useful in uncovering the nature of the chemical changes evidenced by the spectroscopic data and in identifying outliers. Two trends could be identified with PCA, one related to the drop in *M*_w_ and PD and the other related to an increased saturation (e. g., as a result of hydrogenation of the aromatic bonds present in treated kraft lignin). A comparison of models showed that future predictions are best obtained with non‐logarithmic values for *M*_w_ and PD. The obtained results show that operando ATR‐IR spectroscopy combined with multivariate regression can provide quantitative results that are comparable to more traditional SEC measurements and thus can ultimately replace these time‐consuming SEC measurements. Indeed, the developed ATR‐IR/PLS model can be integrated and automated, ultimately offering the possibility to apply it for online process monitoring and control of lignin‐based conversion processes.

## Experimental Section

Materials: Pt/γ‐Al_2_O_3_ catalyst was obtained from Sigma‐Aldrich and used as received. Indulin AT kraft lignin (originating from softwood) was obtained from a commercial supplier (Ingevity, USA).

IR spectroscopy: ATR FT‐IR spectra of the reaction were recorded on a MT ReactIR 45 m ATR‐IR spectrometer equipped with an MCT detector using a bottom mounted ATR Sampling Accessory with a diamond/ZnSe plate. The spectra were collected in the range 4000–650 cm^−1^ using Mettler Toledo iC IR 4.2 software with 1 min acquisition time, 128 scans per sample, and 4 cm^−1^ spectral resolution. The APR reaction was conducted in a bottom‐mounted ATR‐IR equipped 100 mL Parr autoclave. For this purpose, 14.0 g kraft lignin was dissolved in a 56.0 mL alkaline aqueous solution (3.5 wt% NaOH, pH=13.58), while 4.62 g of the Pt/Al_2_O_3_ catalyst was used to promote the catalytic reaction. APR was conducted at 225 °C and 30 bar He pressure for 320 min, while the pressure was controlled by a back‐pressure regulator and sampling from the reactor was performed using a custom‐built sampling line that allowed sampling at high pressure and temperature. The reactor was loaded with the above reaction mixture, purged three times with He, and pressurized to 30 bar and then heated to 225 °C. The FT‐IR spectra were acquired starting from room temperature up to the set temperature and continued till the end of the APR reaction. After catalysis, the autoclave was air cooled. The single‐beam reaction spectra were reconstructed from the absorption reaction spectra and the background spectrum used at the beginning of the experiment. In a separate set of experiments to acquire the solvent spectra at the same pH value as in the reaction run, 70 g of the aqueous alkaline solvent (2.7 wt% NaOH, pH=13.58) was heated in the ATR‐IR bottom‐mounted 100 mL Parr autoclave from room temperature to 225 °C under 30 bar He pressure. The temperature‐resolved absorption ATR‐IR spectra of the solvent were acquired using optimum flushing time of at least 2.5 h with 1 min acquisition time, 128 scans per sample, and 4 cm^−1^ spectral resolution. The single‐beam solvent spectra were reconstructed from the absorption solvent spectra and the background spectrum used at the beginning of the experiment.

SEC: *M*_w_ and PD of the samples taken from the APR reaction mixture were determined by SEC as published by Constant et al. as SEC method D.^[13]^ Molar mass determination was performed on an alkaline SEC using a Waters Alliance instrument equipped with a TSK‐gel guard column PWxl, column size: 6.0 mm I.D.×4 cm, particle size: 12 μm, and two serial connected columns packed with TSK‐gel GMPWxl, column size: 7.8 mm I.D.×30 cm, particle size: 13 μm. Sodium polystyrene sulfonates with *M*_w_ range of 891–976000 Da were used for the calibration of the molar mass distribution. The APR reaction samples were diluted to a concentration of 1 mg mL^−1^ lignin in 0.5 m NaOH. SEC runs were performed at 30 °C with 0.5 m NaOH eluent at a flow rate of 1 mL min^−1^ and UV detection at 280 nm. The APR samples were measured in duplicate.

GC and GC‐MS: For the GC and GC‐MS analyses, 0.5 mL sample was passed over 0.5 g of silica gel padded with 0.2 g of magnesium sulfate to remove water and the inorganic compounds, while the organics were subsequently eluted with 3 mL ethyl acetate. 100 μL (0.032 mmol) mesitylene was added to the eluent, which was then analyzed by GC and GC‐MS. GC measurements were performed on a Varian GC instrument equipped with a VF‐5 ms capillary column and a flame ionization detector (FID), while GC‐MS measurements were performed on a Shimadzu GC‐2010 instrument using a VF5‐ms column, coupled to a Shimadzu GCMS‐QP2010 mass spectrometer.

Multivariate analysis: The multivariate analysis was done using the PLS Toolbox developed by Eigenvector Research, Inc. The PLS Toolbox is an advanced chemometric analysis tool for use within the MATLAB® computational environment. For each regression case, the property of interest (*M*_w_ or PD) was modelled using a separate PLS model for each property. The ATR‐IR spectra were normalized with MSC and then mean‐centered, while the *M*_w_ and PD values were mean‐centered only. Furthermore, the minimization of RMSECV along with the improved explained variance (≈<2 % improvement) were the criteria to decide the number of LVs.

## Conflict of interest

The authors declare no conflict of interest.

## Supporting information

As a service to our authors and readers, this journal provides supporting information supplied by the authors. Such materials are peer reviewed and may be re‐organized for online delivery, but are not copy‐edited or typeset. Technical support issues arising from supporting information (other than missing files) should be addressed to the authors.

Supporting Information
