# Supplementary material for: Monitoring Molecular Weight Changes during Technical Lignin Depolymerization by Operando Attenuated Total Reflectance Infrared Spectroscopy and Chemometrics
Source: ChemSusChem. 2021 Nov 23;14(24):5517–24. doi: 10.1002/cssc.202101853 (PMC9299174; doi:10.1002/cssc.202101853)
Supplement: Supplementary file 1 — Supporting Information [file CSSC-14-5517-s001.pdf]

ChemSusChem

Supporting Information

# Monitoring Molecular Weight Changes during Technical Lignin Depolymerization by Operando Attenuated Total Reflectance Infrared Spectroscopy and Chemometrics

Khaled N. M. Khalili, Peter de Peinder, Jacqueline Donkers, Richard J. A. Gosselink, Pieter C. A. Bruijnincx,* and Bert M. Weckhuysen*© 2021 The Authors. ChemSusChem published by Wiley-VCH GmbH. This is an open access article under the terms of the Creative Commons Attribution License, which permits use, distribution and reproduction in any medium, provided the original work is properly cited.

Figure S1: Parr autoclave equipped with bottom mounted ATR-IR probe with zooming on the attenuated total reflection zone. P: pressure gauge; T: temperature gauge; Vb: back pressure regulator; S1: Sampling valve 1; S2: sampling valve 2; C: clamp; H: heater; I: insulation material; M: mirror. E: evanescent wave; S: total reflection element (sensor); F: focusing element **3**

Figure S2: (A) The single-beam ATR-IR spectra for the 19 samples acquired throughout the Aqueous Phase Reforming (APR) reaction for kraft lignin; (B) The temperature-resolved single-beam ATR-IR spectra of the solvent at the exact same temperatures of sampling **4**

Figure S3: The loadings of the 1$^{st}$ Principal Component (PC1) and 2$^{nd}$ Principal Component (PC2), which explained 95.82% of the spectral changes during the Aqueous Phase Reforming (APR) reaction, in which the important spectral features are highlighted and assigned. **5**

Figure S4: Gas chromatographic-mass spectrometry (GC-MS) chromatogram of the post reaction sample of the Aqueous Phase Reforming (APR) reaction of kraft lignin. The reactions were conducted over a $Pt/Al_2O_3$ catalyst with 3.5 wt.% NaOH in a 100 ml Parr autoclave equipped with a bottom-mounted ATR-IR accessory. **5**

Figure S5: (A) Performance of Partial Least Square (PLS) Model A upon prediction of average-weight Molecular Weight ($M_w$) along with important statistics. (B) Performance of PLS Model B upon prediction of Polydispersity (PD) along with important statistics. LV = Latent Variable; RMSEP = Root Mean Squared Error of Prediction; RMSECV = Root Mean Squared Error of Cross-Validation; Pred. bias = Prediction Bias; and $R^2$ (Pred)= coefficient of determination for the prediction. **6**

Figure S6: (A) Performance of Partial Least Square (PLS) Model C upon prediction of average-weight Molecular Weight ($M_w$) along with important statistics. (B) Performance of PLS Model C upon prediction of Polydispersity (PD) along with important statistics. LV = Latent Variable; RMSEP = Root Mean Squared Error of Prediction; RMSECV = Root Mean Squared Error of Cross-Validation; Pred. bias = Prediction Bias; and $R^2$ (Pred)= coefficient of determination for the prediction. **7**

Figure S7: The regression vectors associated with the best PLS Model, i.e., model B in Table 1, to predict (A) Mw; and (B) PD **8**

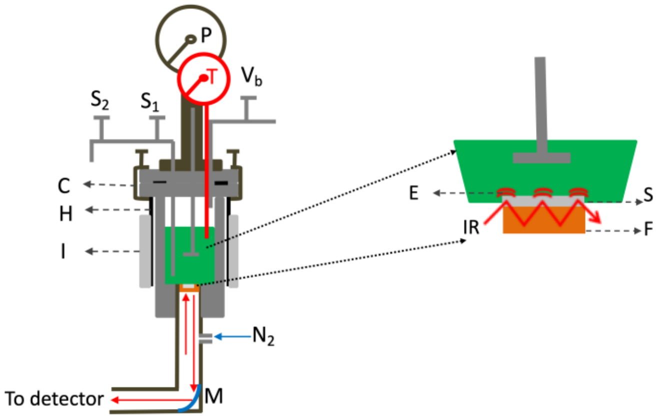


Figure S1: Parr autoclave equipped with bottom-mounted ATR-IR probe with zooming on the attenuated total reflection zone. P: pressure gauge; T: temperature gauge; $V_b$: back pressure regulator; S1: Sampling valve 1; S2: sampling valve 2; C: clamp; H: heater; I: insulation material; M: mirror. E: evanescent wave; S: total reflection element (sensor); F: focusing element

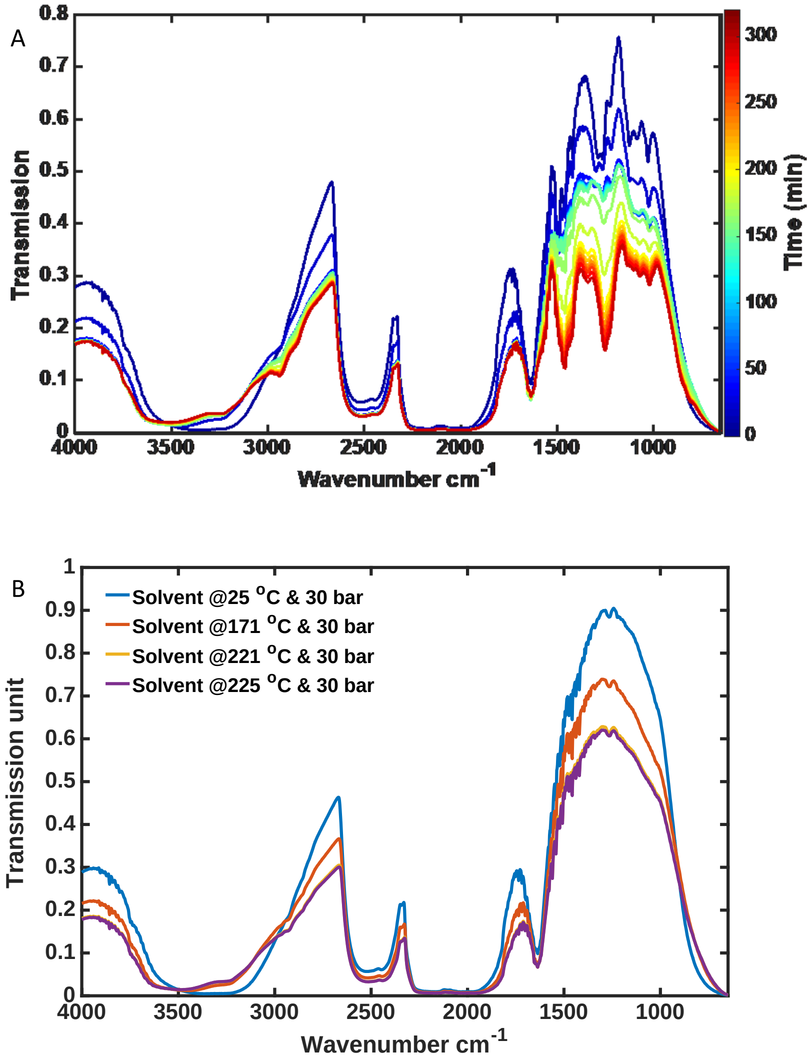


Figure S2: (A) The single-beam ATR-IR spectra for the 19 samples acquired throughout the Aqueous Phase Reforming (APR) reaction for kraft lignin; (B) The temperature-resolved single-beam ATR-IR spectra of the solvent at the exact same temperatures of sampling.

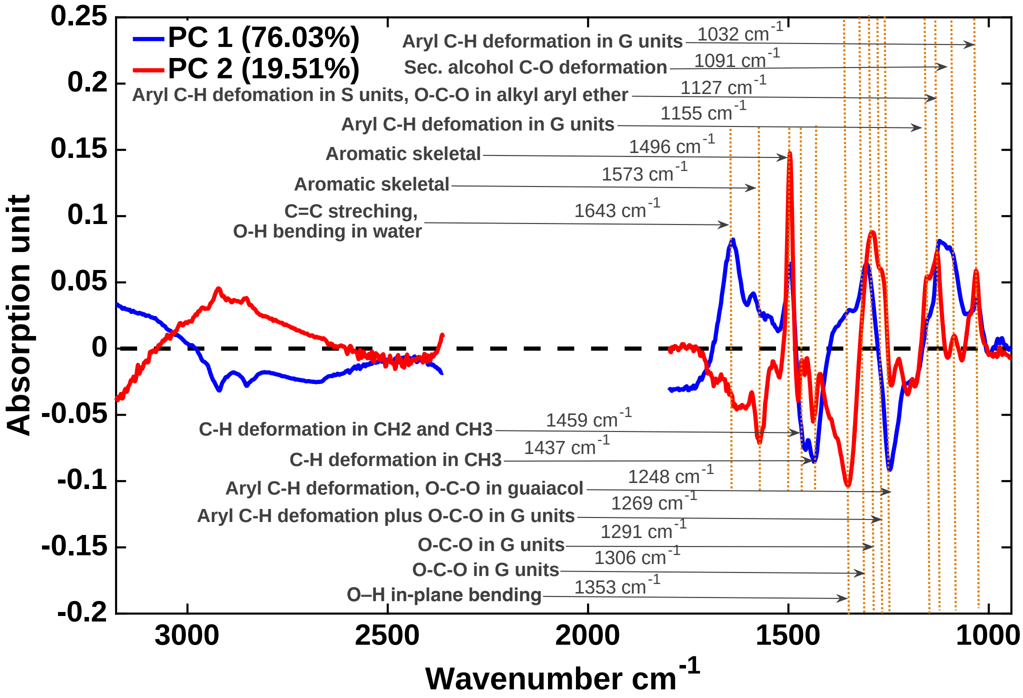


Figure S3: The loadings of the 1st Principal Component (PC1) and 2nd Principal Component (PC2), which explained 95.82% of the spectral changes during the Aqueous Phase Reforming (APR) reaction, in which the important spectral features are highlighted and assigned.

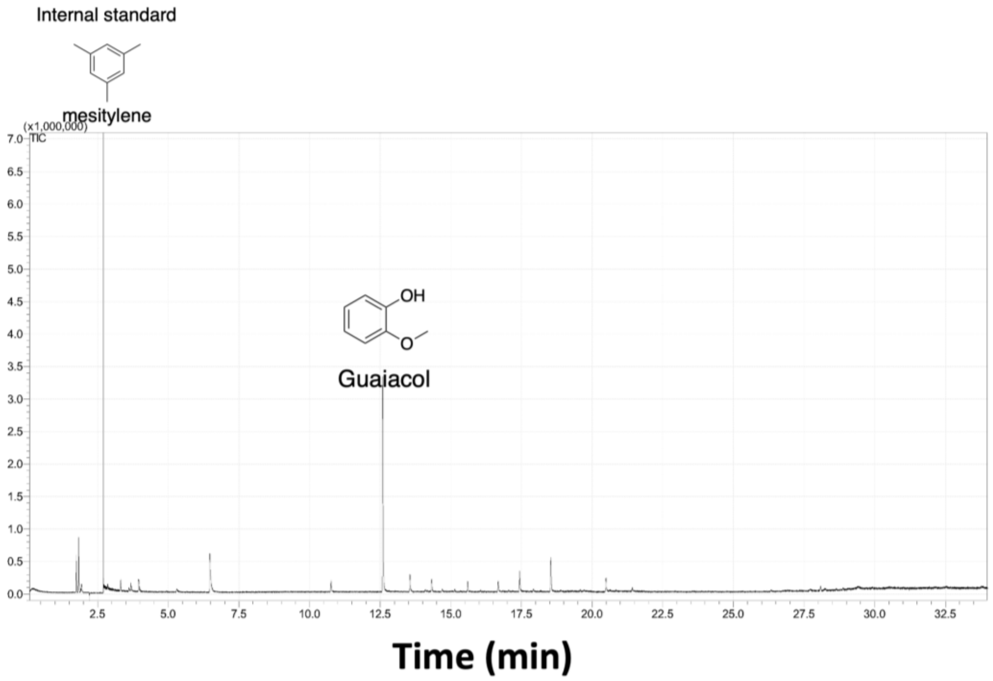


Figure S4: Gas chromatography-mass spectrometry (GC-MS) chromatogram of the post reaction sample of the Aqueous Phase Reforming (APR) reaction of kraft lignin. The reactions were conducted over a $Pt/Al_2O_3$ catalyst with 3.5 wt.% NaOH in a 100 ml Parr autoclave equipped with a bottom-mounted ATR-IR accessory.

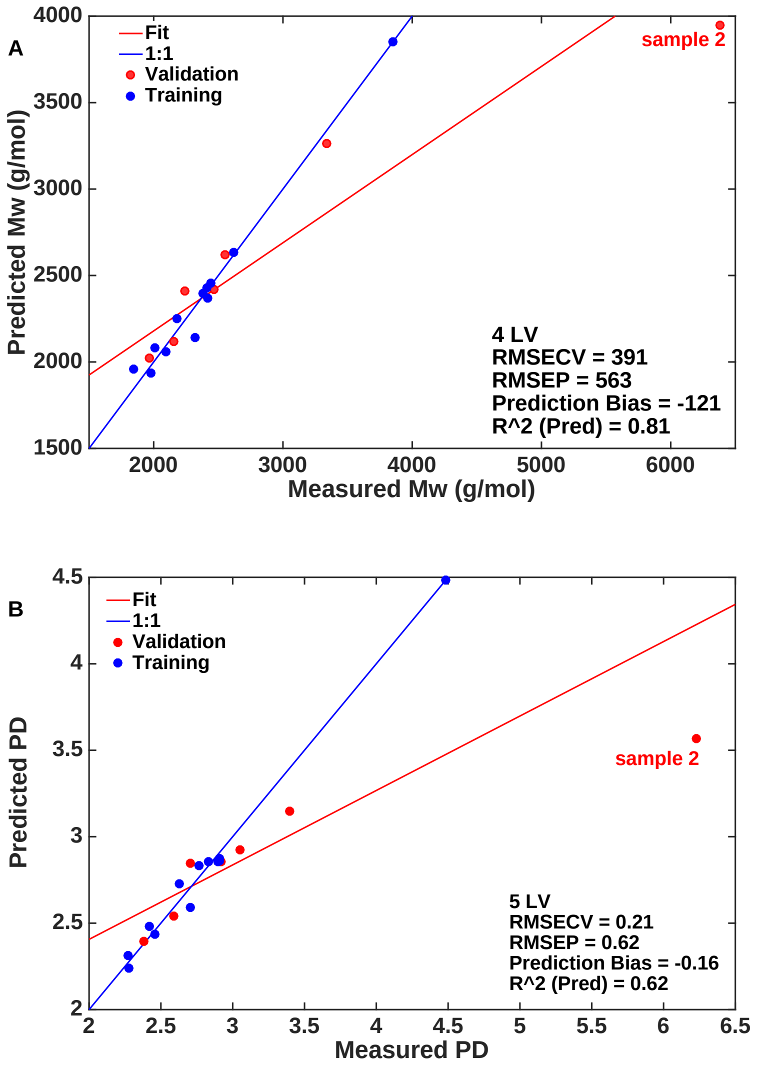


Figure S5: (A) Performance of Partial Least Square (PLS) Model **A** upon prediction of weight average Molecular Weight ($M_w$) along with important statistics. (B) Performance of PLS Model B upon prediction of Polydispersity (PD) along with important statistics. LV = Latent Variable; RMSEP = Root Mean Squared Error of Prediction; RMSECV = Root Mean Squared Error of Cross-Validation; Pred. bias = Prediction Bias; and $R^2$ (Pred)= coefficient of determination for the prediction.

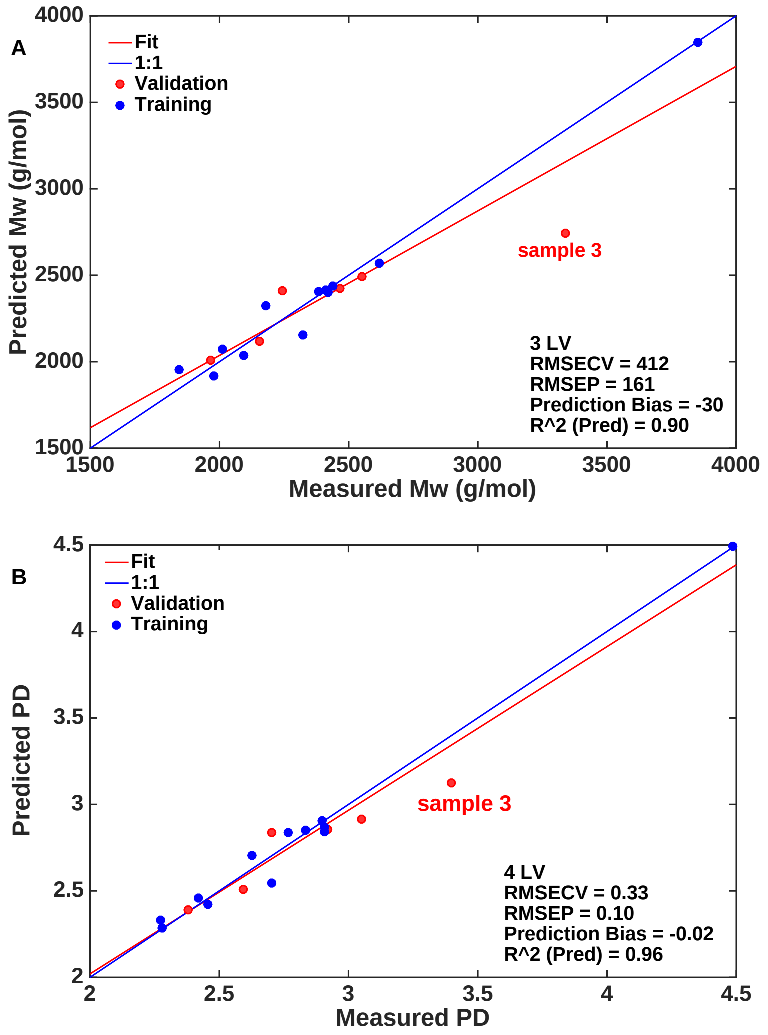


Figure S6: (A) Performance of Partial Least Square (PLS) Model **C** upon prediction of weight average Molecular Weight ($M_w$) along with important statistics. (B) Performance of PLS Model C upon prediction of Polydispersity (PD) along with important statistics. LV = Latent Variable; RMSEP = Root Mean Squared Error of Prediction; RMSECV = Root Mean Squared Error of Cross-Validation; Pred. bias = Prediction Bias; and $R^2$ (Pred)= coefficient of determination for the prediction.

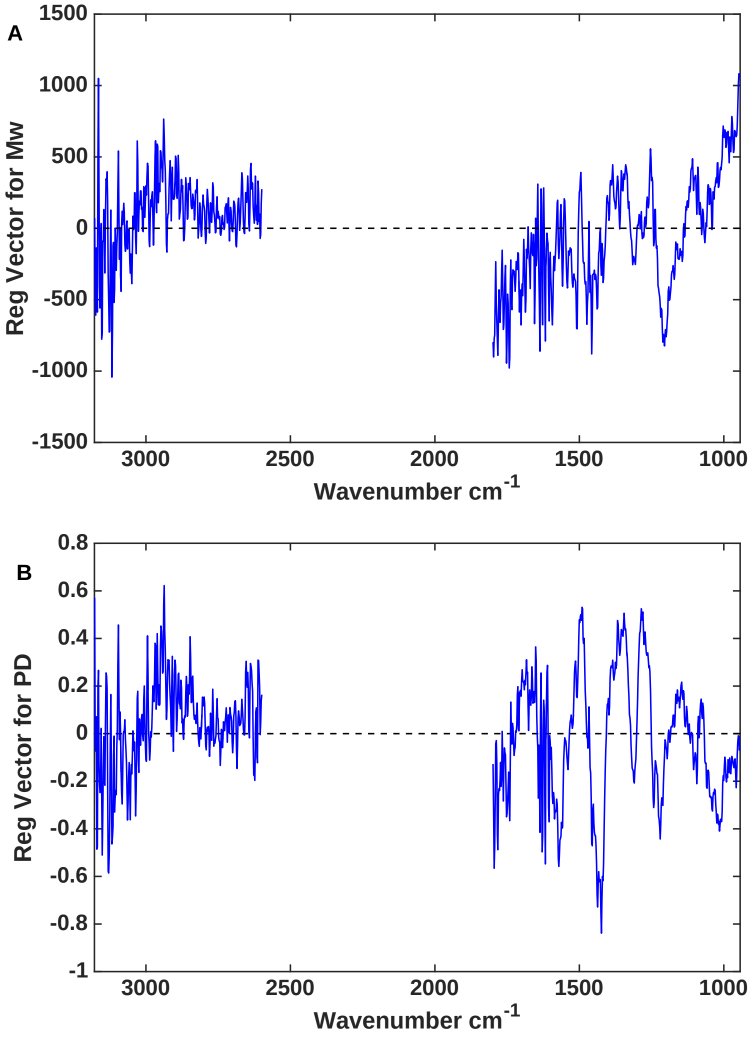


Figure S7: The regression vectors associated with the best PLS Model, i.e., model **B** in Table 1, to predict (A) Mw; and (B) PD.
